# The effect of a combined long‐duration static stretching and resistance training regimen on a competitive bodybuilder: A case study

**DOI:** 10.14814/phy2.70156

**Published:** 2025-01-17

**Authors:** Kai A. Homer, Eric R. Helms, Alyssa‐Joy Spence

**Affiliations:** ^1^ Sport Performance Research Institute New Zealand (SPRINZ) Auckland University of Technology Auckland New Zealand; ^2^ Department of Exercise Science and Health Promotion, Muscle Physiology Laboratory Florida Atlantic University Boca Raton Florida USA

**Keywords:** case report, chronic stretching, muscle architecture, range of motion, resistance training

## Abstract

Both resistance training (RT) and long‐duration, high‐intensity stretching induce muscular adaptations; however, it is unknown whether the modalities are complementary or redundant, particularly in well‐trained individuals. A case‐study was conducted on a competitive bodybuilder implementing long‐duration, high‐intensity stretching of the plantar flexors (60 min 6x/week for 12 weeks) in conjunction with their habitual RT. Ultrasound muscle architecture (muscle thickness [MT], fascicle length [FL], and pennation angle [PA]) measurements were collected at multiple sites at four weekly baseline sessions, six (mid) and 12 (post1) weeks following the commencement of the intervention, and a week after the intervention (post2) while isometric strength and range of motion (RoM) were obtained once at baseline, mid, post1, and post2. 2SD band plots were constructed to determine meaningful changes in MT, FL, and PA from the four baseline measures while percentage and absolute change across each timepoint were calculated for all variables. From baseline to post 1, RoM, strength, and MT increased 25.9%, 11.4%, and 7.4%–23.4%, respectively, while four MT and two PA sites exceeded the threshold for meaningful change. The combined stretching and RT protocols resulted in flexibility, strength, and MT adaptations; however, findings should be generalized with caution given the case‐study nature of our investigation.

## 1 INTRODUCTION

Recently, Warneke, Brinkmann, et al. (2022) observed significant gastrocnemius muscle thickness (MT) increases (~15%) following 6 weeks of daily, long‐duration (60 min), high‐intensity (8/10 on a pain scale) stretching using an orthotic device in active participants. Additionally, high‐frequency (5×/week), progressive stretching of the plantar flexors in male participants with unknown training histories on a leg press for 6weeks for only 3 min each session – resulted in smaller MT increases (~6%) (Simpson et al., 2017) with unclear differences compared to non‐stretched limbs (Jakobi et al., 2018; Nunes et al., 2018). Therefore, to induce notable hypertrophy, high‐frequency stretching likely must be long‐duration and high‐intensity, particularly as a dose–response relationship may exist (Warneke et al., 2024).

Subsequently, Warneke, Wirth, et al. (2023) compared the same six‐week intervention to three times per week progressive resistance‐training (RT) in active participants. Both protocols caused significant increases in ankle dorsiflexion range of motion (RoM), gastrocnemius MT, gastrocnemius lateralis pennation angle (PA), and strength; however, there were no significant between‐group differences. Thus, while both RT and long‐duration, high‐intensity stretching produce muscular adaptations, concurrently performing both types of training could prove complementary or redundant.

Stretching interventions have previously been conducted on participants considered to be trained in sporting contexts but not with stretch training (Panidi et al., 2021; Warneke, Keiner, et al., 2023; Wilson et al., 1992). However, no continuous long duration static stretching (>30 min per muscle per session [Warneke, Lohmann, et al., 2023]) data exists in individuals with extensive RT histories such as bodybuilders, and it is unknown whether such stretching interventions can potentiate muscular adaptation in such populations. Therefore, we conducted a case‐study on a competitive bodybuilder not previously exposed to stretch training using a similar plantar flexor orthotic stretching device as used in previous research (Warneke, Brinkmann, et al., 2022) with concurrent RT. We aimed to determine, preliminarily, if this combination could induce superior RoM, strength, and muscle size and architecture adaptations compared to RT alone.

## 2 METHODS

### 2.1 Participant information

A drug‐free, male, 39‐year‐old competitive bodybuilder (height = 185 cm; body mass = 95 kg) with 18 years RT experience completed this case study. The Auckland University of Technology Ethics Committee previously approved (AUTEC reference number 12/332) Sport Performance Research Institute New Zealand (SPRINZ) staff members to conduct research on themselves and one another—with consent—and the participant in this study was both a SPRINZ staff member and an author (ERH) on this paper, who provided informed consent to participate. The study conformed to the ethical requirements of AUTEC and to the Code of Ethics of the World Medical Association (Declaration of Helsinki).

### 2.2 Timeline

Seven testing sessions were completed by the participant in the laboratory. The first four sessions took place at the same time, 1 week apart to determine baseline ultrasound muscle architecture values. The fourth session consisted of a full pre‐test (pre); following sonography, RoM was tested using the knee‐to‐wall test, then maximal isometric plantar flexor strength. Mid‐testing (mid) was completed after 6 weeks, the post‐test (post1) after another 6 weeks, then a follow‐up (post2) 1 week later. Testing protocols were the same at pre, mid, post1, and post2. Finally, the CARE Case Report Guidelines were used to guide the preparation of this manuscript.

### 2.3 Outcome assessments

#### 2.3.1 Knee‐to‐wall test

A measuring tape was secured at the junction of wall and floor. The participant placed their right foot on the tape with a stretched rubber band under their heel, then flexed the knee to touch the wall (Figure S1). Attempts were successful if the knee touched the wall with the rubber band in place. After successful attempts, the foot was moved farther from the wall. A maximum of five attempts was allowed with the largest value recorded.

#### 2.3.2 Maximal voluntary isometric contraction

A dip belt attached to a strain gauge via a chain was worn by the participant, holding them in position (Figure S2). The same chain length was used session to session to standardize ankle joint position, which constrained the participant from reaching maximal plantarflexion. The participant was instructed to raise their heels, taking the slack out of the chain, and push as hard as possible with verbal encouragement provided until force production plateaued. Three trials, separated by 3 min rest, were completed each session with peak values recorded.

#### 2.3.3 Ultrasonography

Muscle architecture values were obtained with two‐dimensional B‐mode ultrasonography with the L12‐4, linear‐array 37 mm probe (Lumify; Philips Healthcare, Amsterdam, Netherlands) on the Musculoskeletal setting connected to a Samsung Galaxy Tablet S7 FE (Samsung, Seoul, South Korea) using the Lumify application (version 4.04). This probe was validated for muscle architecture and MT measurements against stationary, high‐end laboratory devices (Homer, Cross, & Jukic, 2024; Ritsche et al., 2022). The participant lay prone with their feet over a table edge to ensure relaxation of the musculature. Measurements were collected one third of the distance from the proximal tibia to the base of the medial malleolus at mid‐muscle belly for lateral and medial heads. Proximal and distal measurements were taken 4 cm from the central marker for the medial head. Different regions were assessed based on previous recommendations to increase the validity of measurements (Nunes et al., 2023; Wohlann et al., 2024) as well as to discern any inhomogeneous changes in response to the intervention.

Water‐soluble transmission gel was applied to the probe which was positioned with the least pressure required to obtain images. Images were captured in triplicate, in the transverse plane for the medial and lateral plantar flexors and longitudinally for other sites to visualize muscle architecture. For the four sites where muscle architecture measurements were obtained (middle gastrocnemius medialis and lateralis, proximal and distal gastrocnemius medialis), the probe was rotated slowly until the superficial and deep aponeuroses were parallel and the fascicles were clearly visualized (Warneke, Wirth, et al., 2023). The researcher possessed previous experience collecting muscle ultrasound measurements using the Lumify probe (Homer et al., 2024).

All images were analyzed in ImageJ (Java 1.8.0_172) by the same researcher. MT was determined as the linear vertical distance from the apex of the periosteum to the deep fascia superficial to the muscle and deep to the subcutaneous layer for transverse plane images. Longitudinal plane images were assessed using the semi‐automated SMA tool—which was validated against manual measurements of muscle architecture—to calculate the mean distance between the two aponeuroses of the superficial muscle and extrapolate FL and PA from the dominant fascicles of each image (Seynnes & Cronin, 2020). SMA tool measurements were visually inspected for erroneous results, with parameters adjusted for any subsequent re‐measurements. The mean of at least two images were calculated for analysis.

### 2.4 Intervention

During the first 4 weeks the participant completed RT—including 20 sets of straight knee calf raises per week, performing four sets on each of the 5 days they trained—without stretching. These sets were performed across three exercises (single‐leg bodyweight calf raises, bilateral standing machine calf raises, and bilateral straight leg calf raises on a 45‐degree leg press) between two repetitions‐in‐reserve to momentary failure (i.e., attempting one more repetition after concentric failure) within an 8–40 repetition range. For the following 12 weeks, the same RT regimen was continued and progressed in conjunction with stretching of the plantar flexors for one hour, six days per week.

Stretching was completed on both sides using an orthotic device (Night Splint Dorsal Soft Light; H&F) which placed the ankle joint into dorsiflexion and was adjusted to illicit an 8/10 pain level to start each set of stretching (Figure S3). Testing sessions occurred 24 h after the participant performed a RT session including calf raises to reduce the influence of muscle swelling on measurements. In the final week, the participant lowered RT volume from 20 to eight weekly sets (performing two rather than five sessions) without stretching.

### 2.5 Statistical analysis

Analyses and visualization of MT measurements were performed in R (version 4.3.2). Means and standard deviations for all baseline MT, FL, and PA measurements were calculated, with subsequent measurements deemed meaningful changes from baseline if they were two standard deviations greater or smaller than the baseline average. 2SD band graphs were constructed for MT, FL, and PA measurements while percentage changes were calculated for outcome measures. The dataset and code are available at the Open Science Framework repository (https://osf.io/mp5z2/).

## 3 RESULTS

Ankle dorsiflexion RoM increased each session (13.5, 16, 16.8, 17 cm, total change = +25.9%) with the greatest change occurring pre‐ to mid (+18.5%). Strength increased pre‐ to mid to post1 and decreased in post2 (2156.5, 2283.5, 2402.4, 2349.6 N) where the greatest change (pre‐ to post1) was +11.4%.

**FIGURE 1:**
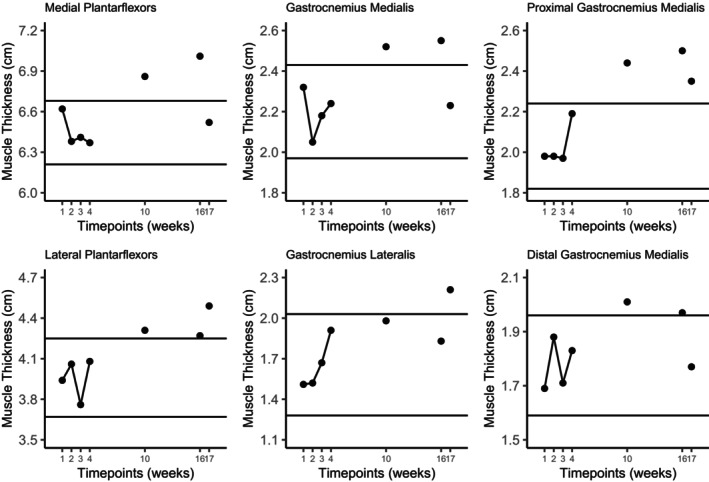
Two standard deviation band graphs of muscle thickness measurements of the posterior lower leg. The horizontal lines indicate the mean ± 2SD of the baseline measurements and represent the threshold for meaningful change.

**FIGURE 2:**
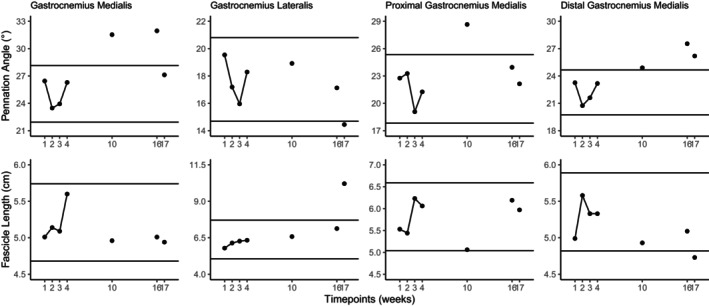
Two standard deviation band graphs of pennation angle and fascicle length measurements of the gastrocnemius. The horizontal lines indicate the mean ± 2SD of the baseline measurements and represent the threshold for meaningful change.

MT of the medial and lateral plantar flexor, proximal medial gastrocnemius, medial gastrocnemius, and distal medial gastrocnemius MT increased 7.4%, 7.7%, 23.4%, 17.9%, 11.2% respectively (Figure 1), while PA increased at the medial gastrocnemius (+27.6%) and distal medial gastrocnemius (+24.1%) from averaged baseline measurements to post1. No meaningful change occurred in lateral gastrocnemius MT, proximal medial gastrocnemius and lateral gastrocnemius PA, as well as FL at each site (Figure 2) in accordance with the predetermined 2SD threshold.

## 4 DISCUSSION

This case report investigated the effect of a 12‐week, 1 h daily stretching protocol using an orthotic device on plantar flexor flexibility, strength, and muscle architecture of a competitive bodybuilder. While increased strength, flexibility, and MT has been previously observed in active individuals (Warneke, Brinkmann, et al., 2022; Warneke, Keiner, et al., 2023; Warneke, Lohmann, et al., 2022; Warneke, Wirth, et al., 2023) we report these outcomes in an experienced bodybuilder performing concurrent RT and long‐duration stretching.

Strength increased 5.9% in 6 weeks compared to 9.4%–19.4% in previous works of the same duration (Warneke, Brinkmann, et al., 2022; Warneke, Keiner, et al., 2023; Warneke, Lohmann, et al., 2022) despite the fact that all study populations were untrained in relation to long duration stretching. The difference may be due to the different RT status of participants, as the rate of strength adaptations decrease with increased RT history (Latella et al., 2024). (Warneke, Brinkmann, et al., 2022; Warneke, Keiner, et al., 2023; Warneke, Wirth, et al., 2023) included trained and active individuals, defined as performing two or more training sessions for the previous 6 months, whereas our participant was training 4–6 sessions per week for 18 years.

Despite the expected and often observed RoM improvements, it is difficult to speculate on mechanisms when considering the lack of FL adaptations. While chronic stretching has been found to increase serial sarcomere number within animals (Warneke, Wirth, et al., 2023) which may contribute to increased RoM; FL, a proxy for serial sarcomere number, did not meaningfully increase across the present study. This is in line with previous research which reported no increases in FL despite enhanced RoM following long‐term stretching protocols (>12 weeks) (Longo et al., 2021; Moltubakk et al., 2021; Panidi et al., 2021). Thus, it could be inferred that changes in flexibility are influenced more by reductions in pain sensitivity and changes in connective tissue properties than muscle architecture adaptations (Freitas et al., 2018; Warneke, Wirth, et al., 2023).

Region‐specific PA changes were found within the middle and distal portions of the gastrocnemius medialis. These findings are similar to Warneke, Wirth, et al. (2023) who found significant PA increases in the middle of gastrocnemius medialis but not in the lateralis; however, these results were similar in both stretching and RT groups. Somewhat contrastingly, Simpson et al. (2017) reported decreased PA to account for increased FL, particularly in the gastrocnemius lateralis. Based on our predetermined threshold, PA in the gastrocnemius lateralis did not decrease from baseline at mid‐point or post1, which may be due to near maximal architectural adaptations at this site in our participant from years of RT. This is plausible given that RT elicited similar adaptations to stretch training in the study by Warneke, Wirth, et al. (2023).

In the present study, gastrocnemius MT increases in the medial and lateral head after 6 weeks were larger than that observed by (Warneke, Brinkmann, et al., 2022; Warneke, Keiner, et al., 2023; Warneke, Wirth, et al., 2023) which may be due to concurrent stretching and RT. While MT values were variable, any measurable hypertrophy occurring over a relatively short period in a highly trained athlete is arguably notable. Theoretically, the underlying primary mechanism of stretch‐induced hypertrophy—like RT‐induced hypertrophy—is thought to be mechanical tension (Warneke, Lohmann, et al., 2023). Therefore, considering measurable hypertrophy occurred, if mechanical tension is the underlying mechanism and primary stimuli, these findings may support the notion that passive and active tension stimulate different anabolic pathways (Warneke, Lohmann, et al., 2023). Notably, the active and passive mechanosensors in skeletal muscle are seemingly stimulated by different forces (Schoenfeld et al., 2022), potentially making stretching and RT complementary. Alternatively, or together with mechanical tension, other potential complementary mechanisms related to RT or stretching may play a role, such as exercise induced metabolite accumulation (Wackerhage et al., 2019) or hypoxia (Warneke, Lohmann, et al., 2023).

Importantly, these conclusions are speculative, and our investigation is not without limitations. As a case study the present investigation lacks external validity; the results cannot generalize to the broader population or to other bodybuilders following differing training and nutritional practices. We also did not measure the passive and active tension during stretching and RT, respectively, making it difficult to speculate on physiological mechanisms of muscular adaptation. Future research could incorporate the assessment of these variables to further elucidate the influences of active and passive mechanosensors. Importantly, determining meaningful change thresholds in a single‐subject design for measurements with day‐to‐day variation and measurement error is challenging, particularly with the limitations surrounding ultrasound measurements of muscle architecture without the availability of extended field‐of‐view scanning (Franchi et al., 2018). Additionally, we conservatively chose a 2SD change from the average baseline mean instead of 1SD to increase the confidence in observing true changes, potentially elevating the likelihood of type II error. This may be compounded by baseline variation in some measurements upon visual inspection (e.g., gastrocnemius lateralis and proximal gastrocnemius medialis MT in Figure 1), increasing the SD and change required to surpass the threshold. Thus, while we included and interpreted changes across multiple regions of the plantar flexors to increase validity and provide a clearer depiction of the intervention effects (Nunes et al., 2023), it is possible true changes were not detected for measurements at certain sites.

## 5 CONCLUSION

Combined stretching and RT resulted in superior MT adaptations compared to RT alone (baseline) in a competitive bodybuilder; however, FL and PA changes were limited, perhaps indicating regional differences in response to stretch training. Additionally, this protocol resulted in increased flexibility and isometric strength over 12 weeks. Based on these findings, it is worthwhile for trained individuals to experiment with similar interventions and for further research to be conducted in similar populations.

## FUNDING INFORMATION

The authors confirm that no funding was received for the study.

## CONFLICT OF INTEREST STATEMENT

The authors confirm no conflicts of interest nor that there was any relationship with the manufacturer of the device used in the study.

## ETHICS STATEMENT

The Auckland University of Technology Ethics Committee previously approved (AUTEC reference number 12/332) Sport Performance Research Institute New Zealand (SPRINZ) staff members to conduct research on themselves and one another—with consent—and the participant in this study (ERH) was both a SPRINZ staff member and an author on this paper, who provided informed consent to participate in this case study as well as for the publication of the images included as the supplementary figures. The study conformed to the ethical requirements of the Auckland University of Technology Ethics Committee and to the Code of Ethics of the World Medical Association (Declaration of Helsinki).

## Supporting information


Figure S1.

**Figure S2.**.

Figure S3.

## Data Availability

The associated dataset, code, and additional parameters are available in the Open Science Framework repository (https://osf.io/mp5z2/).
