# Supplementary figures and images for: The effect of a combined long‐duration static stretching and resistance training regimen on a competitive bodybuilder: A case study
Source: Physiol Rep. 2025 Jan 17;13(2):e70156. doi: 10.14814/phy2.70156 (PMC11824903; doi:10.14814/phy2.70156)

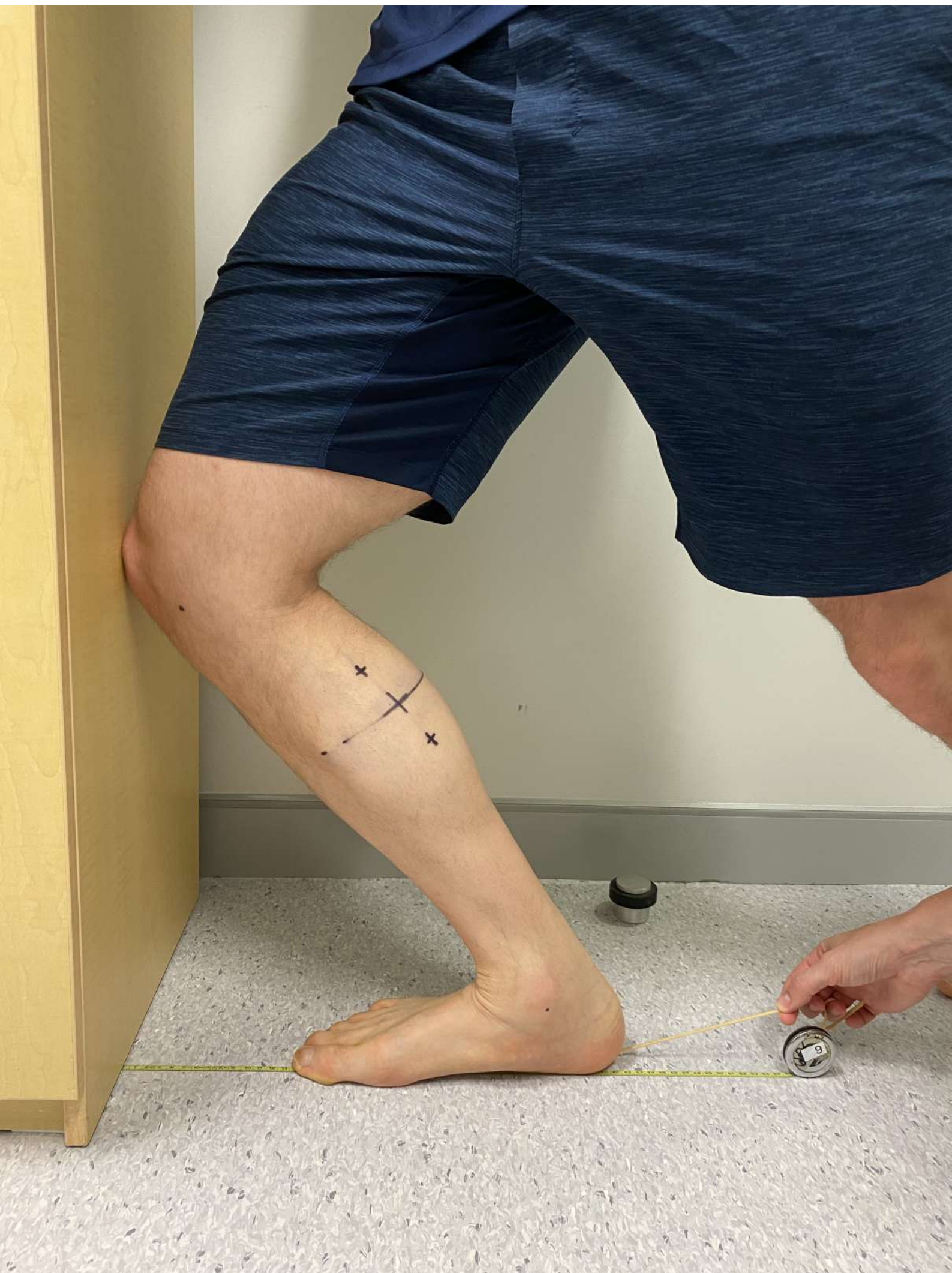

Supplement: Supplementary file 1 — Figure S1. [file PHY2-13-e70156-s001.pdf]

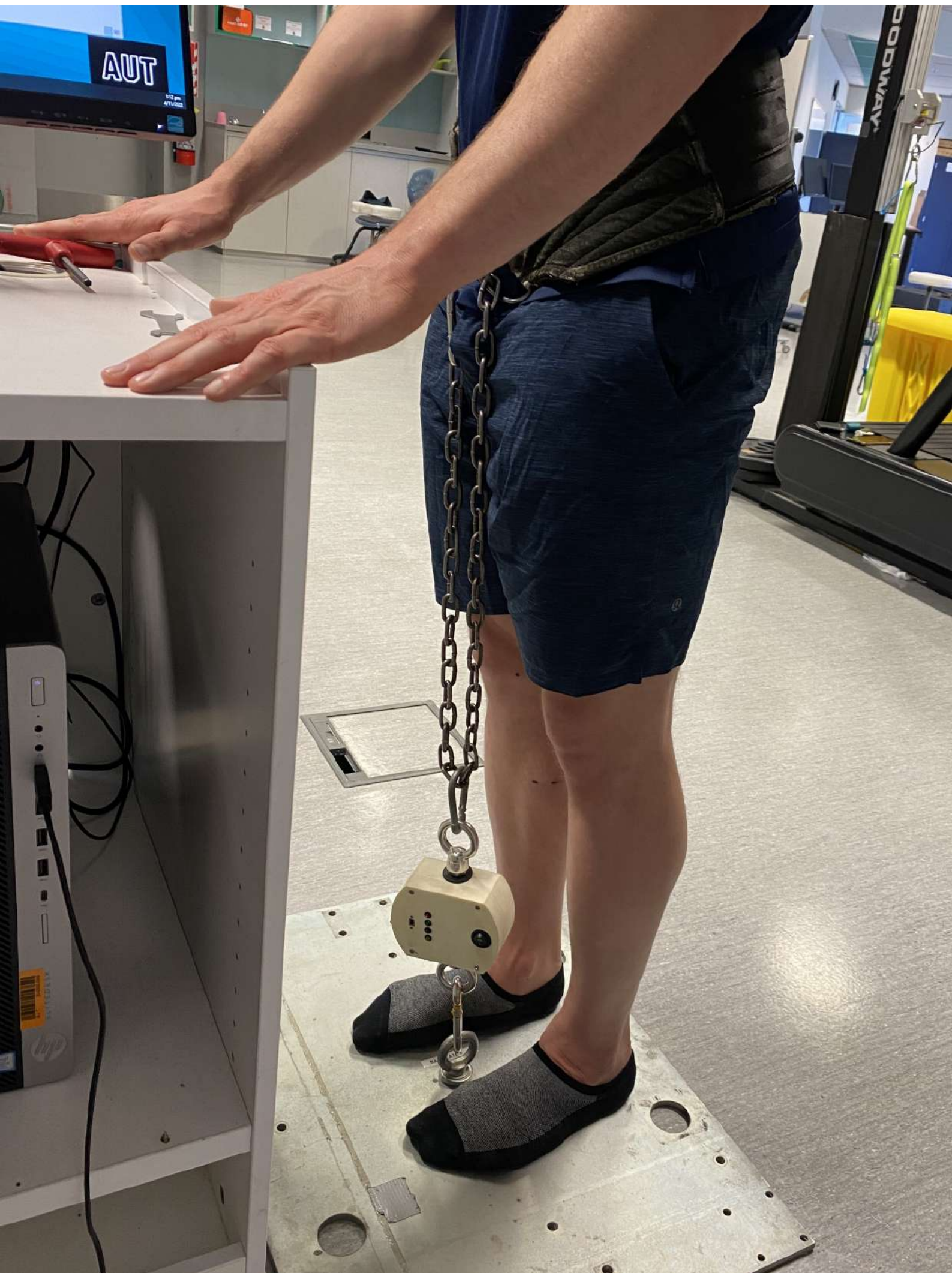

Supplement: Supplementary file 2 — Figure S2.. [file PHY2-13-e70156-s002.pdf]

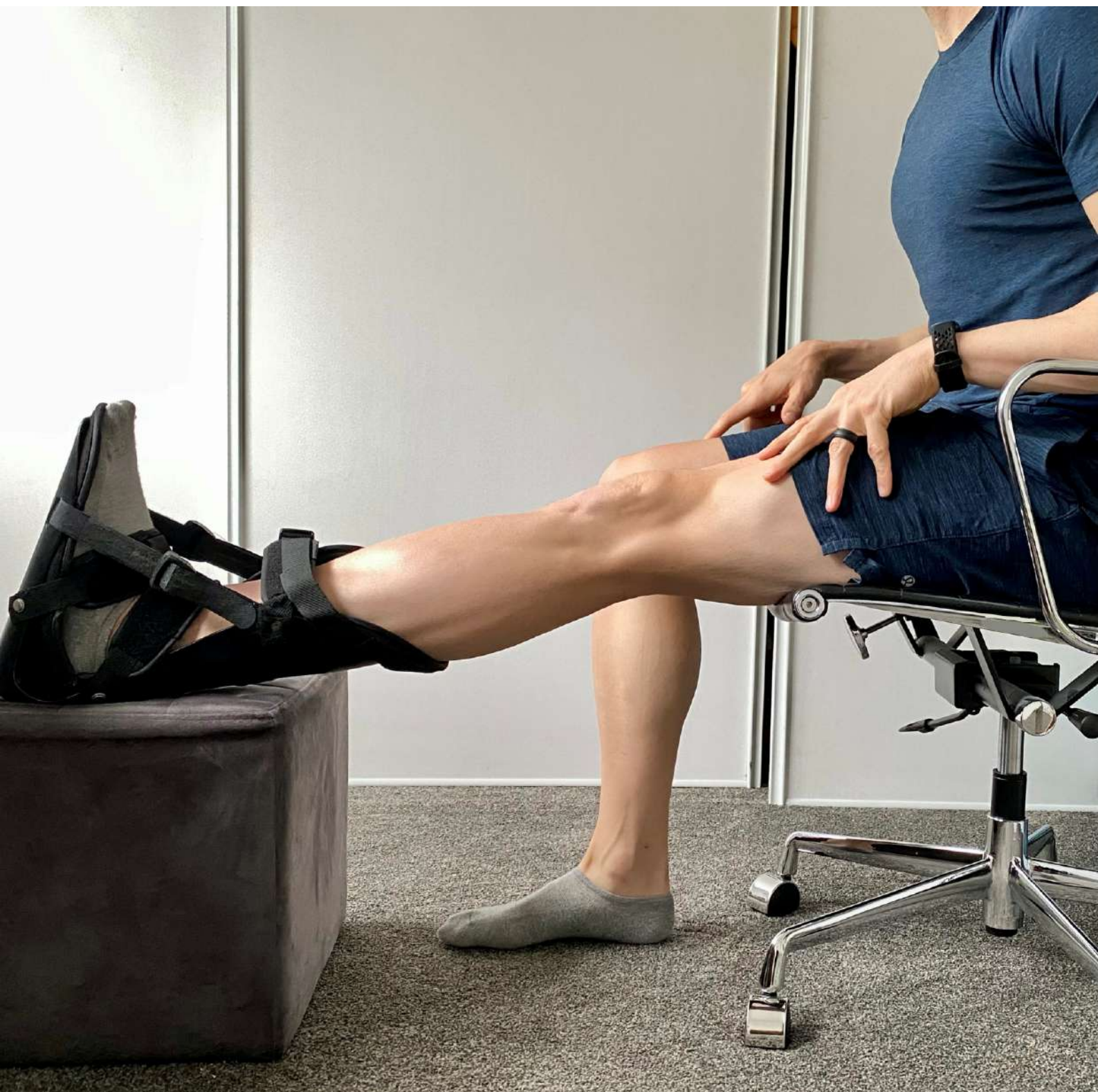

Supplement: Supplementary file 3 — Figure S3. [file PHY2-13-e70156-s003.pdf]
